# The DNA methylome of DDR genes and benefit from RT or TMZ in IDH mutant low-grade glioma treated in EORTC 22033

**DOI:** 10.1007/s00401-018-1810-6

**Published:** 2018-01-24

**Authors:** Pierre Bady, Sebastian Kurscheid, Mauro Delorenzi, Thierry Gorlia, Martin J. van den Bent, Khê Hoang-Xuan, Élodie Vauléon, Anja Gijtenbeek, Roelien Enting, Brian Thiessen, Olivier Chinot, Frédéric Dhermain, Alba A. Brandes, Jaap C. Reijneveld, Christine Marosi, Martin J. B. Taphoorn, Wolfgang Wick, Andreas von Deimling, Pim French, Roger Stupp, Brigitta G. Baumert, Monika E. Hegi

**Affiliations:** 1 Laboratory of Brain Tumor Biology and Genetics, Neuroscience Research Center, Lausanne University Hospital, Chemin des Boveresses 155, CLE-C306, 1066 Epalinges, Lausanne, Switzerland; 2 Division of Neurosurgery, Lausanne University Hospital, Lausanne, Switzerland; 3 Department of Education and Research, Lausanne University Hospital, Lausanne, Switzerland; 4 Bioinformatics Core Facility, SIB Swiss Institute of Bioinformatics, Lausanne, Switzerland; 5 Department of Genome Science, The Australian National University, Canberra, Australia; 6 Department of Oncology, Lausanne University Hospital, Lausanne, Switzerland; 7 Ludwig Center for Cancer Research, University of Lausanne, Lausanne, Switzerland; 8 EORTC Headquarter, Brussels, Belgium; 9 The Brain Tumor Center at Erasmus MC Cancer Institute, Rotterdam, The Netherlands; 10 APHP Pitié-Salpétrière, Sorbonne Universités, UPMC, UMR S 1127, Paris, France; 11 Regional Cancer Institute Eugène Marquis, Rennes, France; 12 Radboud University Medical Center, Nijmegen, The Netherlands; 13 UMCG, University of Groningen, Groningen, The Netherlands; 14 BC Cancer Agency, Vancouver, BC Canada; 15 Aix-Marseille Université, AP-HM, Hôpital de la Timone, Marseille, France; 16 Institut Gustave Roussy, Villejuif, France; 17 Ospedale Bellaria, Bologna, Italy; 18 Brain Tumor Center and Department of Neurology, VU University Medical Center, Amsterdam, The Netherlands; 19 Clinical Division of Medical Oncology, Department of Internal Medicine I, Medical University of Vienna, Vienna, Austria; 20 Haaglanden Medical Center, The Hague, The Netherlands; 21 Clinical Cooperation Unit Neurooncology, German Cancer Consortium (DKTK), German Cancer Research Center (DKFZ), Heidelberg, Germany; 22 Department of Neurology and Neurooncology Program, National Center for Tumor Diseases, Heidelberg University Hospital, Heidelberg, Germany; 23 German Cancer Consortium (DKTK) and CCU Neuropathology German Cancer Research Center (DKFZ), Heidelberg, Germany; 24 Department Neuropathology, Institute of Pathology, University of Heidelberg, Heidelberg, Germany; 25 Malnati Brain Tumor Institute at the Lurie Comprehensive Cancer Center, Northwestern University Feinberg School of Medicine, Chicago, IL USA; 26 Department of Radiation-Oncology (MAASTRO Clinic) & GROW (School for Oncology), Maastricht University Medical Centre, Maastricht, The Netherlands; 27 Department of Radiation-Oncology, Paracelsus Clinic Osnabrück, University of Münster, Münster, Germany

**Keywords:** Low-grade glioma, DNA methylation, TMZ, DDR genes, MGMT-STP27, Randomized trial

## Abstract

The optimal treatment for patients with low-grade glioma (LGG) WHO grade II remains controversial. Overall survival ranges from 2 to over 15 years depending on molecular and clinical factors. Hence, risk-adjusted treatments are required for optimizing outcome and quality of life. We aim at identifying mechanisms and associated molecular markers predictive for benefit from radiotherapy (RT) or temozolomide (TMZ) in LGG patients treated in the randomized phase III trial EORTC 22033. As candidate biomarkers for these genotoxic treatments, we considered the DNA methylome of 410 DNA damage response (DDR) genes. We first identified 62 functionally relevant CpG sites located in the promoters of 24 DDR genes, using the LGG data from The Cancer Genome Atlas. Then we tested their association with outcome [progression-free survival (PFS)] depending on treatment in 120 LGG patients of EORTC 22033, whose tumors were mutant for isocitrate dehydrogenase 1 or 2 (IDHmt), the molecular hallmark of LGG. The results suggested that seven CpGs of four DDR genes may be predictive for longer PFS in one of the treatment arms that comprised *MGMT*, *MLH3*, *RAD21*, and *SMC4*. Most interestingly, the two CpGs identified for *MGMT* are the same, previously selected for the MGMT-STP27 score that is used to determine the methylation status of the *MGMT* gene. This score was higher in the LGG with 1p/19q codeletion, in this and other independent LGG datasets. It was predictive for PFS in the TMZ, but not in the RT arm of EORTC 22033. The results support the hypothesis that a high score predicts benefit from TMZ treatment for patients with IDHmt LGG, regardless of the 1p/19q status. This *MGMT* methylation score may identify patients who benefit from first-line treatment with TMZ, to defer RT for long-term preservation of cognitive function and quality of life.

**Electronic supplementary material:**

The online version of this article (10.1007/s00401-018-1810-6) contains supplementary material, which is available to authorized users.

## Introduction

Optimal treatment strategy of patients with high-risk low-grade glioma WHO grade II remains controversial [45, 46]. The prognosis of patients varies greatly depending on clinical factors (tumor size, patient’s age) and the molecular subtype, oligodendroglioma: mutant for isocitrate dehydrogenase 1 or 2 (*IDH1* or *2*; IDHmt) with co-deletion of the chromosomal arms 1p and 19q (codel); or astrocytoma: with (IDHmt), or without IDH mutation (IDH wild type) [7, 18, 36, 48]. Recent results of the Radiation Therapy Oncology Group trial (RTOG) 9802 phase III trial suggest that early administration of adjuvant chemotherapy [PCV, procarbazine, lomustine (CCNU), and vincristine] following radiotherapy improves overall survival compared to RT alone [8]. Unfortunately, detailed molecular tumor characteristics are not available for the RTOG trial, key information for adequate tumor classification according to the updated WHO classification 2016 [35]. The European Organisation for Research and Treatment of Cancer (EORTC) randomized phase III trial (EORTC 22033) was prospectively designed to compare two treatment modalities, and to identify putative prognostic and predictive molecular markers. Initial clinical results have recently been reported [6]. There was no difference in progression-free survival for patients treated initially with radiotherapy alone or with dose-dense temozolomide [6]. Molecular subgroup analysis according to WHO classification 2016 showed no difference in outcome between the subpopulation of patients with IDHmt codeleted and IDHmt non-codeleted tumors when treated with radiotherapy. In contrast, in the TMZ-treatment arm, patients with IDHmt codeleted tumors did better than the IDHmt and but non-codeleted subgroup. This implies a molecular difference rendering IDHmt codeleted tumors more sensitive to TMZ. This supports previous studies that reported improved responsiveness of 1p/19q co-deleted tumors to therapy; however, at the time IDH mutations were not yet discovered [23].

For individual treatment decisions and risk-adapted therapeutic strategies, it is it is crucial to identify the mechanisms and associated molecular factors predicting benefit from the distinct treatment modalities. For very poor prognosis patients an aggressive and combined modality treatment approach may be warranted, while for patients with a favorable prognosis and long-term survival avoidance of long-term neurocognitive toxicities [14] is of importance.

The majority of low-grade glioma harbor an *IDH1 or 2* mutation, which is associated with a glioma CpG island methylator phenotype (G-CIMP) [16, 39]. This implies that a large number of genes are epigenetically inactivated by promoter methylation, impacting cancer-relevant pathways and potentially modulating treatment response. Given that the patients in EORTC 22033 were randomized to distinct genotoxic treatments, we hypothesized that variations in DNA repair proficiency, associated with G-CIMP linked aberrant promoter methylation, may explain differences in outcome in the two treatment arms. With a focus on IDHmt LGG, we set out to assess the DNA methylome of DNA damage response (DDR) genes that includes *MGMT,* to find predictive factors for treatment. We further aimed at uncovering potential DNA repair vulnerabilities that may be exploited as the “Achilles’ heel” of the tumors, actionable by novel treatment approaches [21].

## Materials and methods

### Patient samples

Tumor specimens (formalin fixed paraffin embedded [FFPE] or frozen) were collected from patients treated in the phase III trial EORTC 22033 (NCT001828199) [6] for high-risk low-grade glioma. Non-tumoral brain tissue (*n* = 5, FFPE) was available from epilepsy surgery. Patients agreed and signed written informed consent for translational research according to institutional and international guidelines and regulations. The trial design of EORTC 22033 comprised a 2-step process: first, patients were registered and tumor tissue was submitted for central pathology review and determination of the deletion status of chromosome 1p (stratification factor). Second, at the time when at least one high-risk feature was present (age > 40 years, progressive disease, tumor size > 5 cm, tumor crossing the midline, or neurological symptoms) patients were randomized to focal radiotherapy (RT, 50.4 Gy in 28 fractions of 1.8 Gy) or dose-dense temozolomide treatment (TMZ, 75 mg/m^2^, 21/28 days, maximum of 12 cycles) [6].

### DNA methylation analysis

For genome-wide DNA methylation analysis, DNA was isolated from macro-dissected tumor tissue (frozen samples: DNeasy Blood & Tissue Kit, Qiagen; FFPE samples: EX-WAX™ Paraffin-embedded DNA Extraction Kit, S4530; Merck KGAa) and quantified (Quant-iT™ PicoGreen^®^ dsDNA Assay Kit, #P7589, Life Technologies). Tumor DNA samples of 150 patients passing a PCR-based quality control (Infinium HD FFPE QC Assay Protocol) were subjected to bisulfite treatment (EZ DNA Methylation-Gold™ Kit, Zymo Research) as previously described [27], and were analyzed on the Human Methylation 450K BeadChip (Illumina, San Diego CA, USA) according to the manufacturer’s protocol at the Genomics platform of the University of Geneva. FFPE samples were analyzed in separate batches after pretreatment with the restoration kit as recommended (Illumina). The dataset is available under the accession number GSE104293 at GEO (http://www.ncbi.nlm.nih.gov/geo/).

### External datasets

External datasets comprised the LGG dataset from The Cancer Genome Atlas (TCGA; *n* = 197; 90 WHO grade II, 106 WHO grade III, 1 unspecified WHO grade) [7] and a set of anaplastic glioma (AGlioma, WHO grade III, *n* = 227; GEO accession number GSE58218) [50]. The TCGA dataset was randomly split into two sub-datasets, called **TCGA-1** and **TCGA-2** (stratified by age, 1p/19q codeletion status, WHO grade, CIMP status, and overall survival), to obtain a test and a validation cohort for marker selection and construction of a predictive model for purity estimation. (dbGaP accession number phs000178.v9.p8; http://cancergenome.nih.gov).

### Data processing

The normalization [20] for Illumina HM-450K arrays included NOOb background correction, dye-correction (chemistry I vs II) and RUV-2 step (removal of unwanted variation) based on control probes using the function *preprocessFunnorm* from the R package minfi [3]. DNA methylation was summarized by *M* values [15]. The ComBat procedure [25] was used to limit experimental variation and batch effects across the four datasets.

### Probe selection

CpG probes with detection *p* values of more than 0.01, located on the sex chromosomes, or in SNPs were removed. A three Gaussian mixture model based on *M* values was used to establish the DNA methylation status of probes in non-tumoral brain tissue (NTB) in order to remove methylated and hemi-methylated probes. Then selection of methylation probes was restricted to CpGs located in gene promoters within 1500 nucleotides up- or down-stream of the transcription start site (TSS). The gene locations were based on Homo sapiens data from UCSC build hg19. The list of DDR genes was adopted from Pearl et al. [41]. A list of 167235 CpGs was detected in promoter regions and 101981 CpGs were considered as unmethylated in non-tumoral brain tissue. CpG methylation was defined as “functional” when the correlation between CpG methylation and expression of the corresponding gene was negative in both datasets TCGA-1 and TCGA-2, with a Pearson correlation coefficient inferior or equal to − 0.3 (corresponds to approximately 10% of explained variance) and an fdr-corrected *p* value not superior to 0.1 for a test of the null hypothesis of correlation equal to 0.

### *MGMT* promoter methylation and the *MGMT* methylation score

The DNA methylation status of the *MGMT* promoter and the *MGMT* score (logit-transformed probability) were determined based on HM-450K data as previously reported [4, 5]. In brief, the *M* values of the methylation probes *cg12434587* and *cg12981137* were used as input into the logistic regression model (MGMT-STP27). A cut-off of 0.358 is used for classification into *MGMT* methylated and unmethylated promoter status, respectively [4, 5].

### Expression

Gene expression from RNAseq data (Level 3) was quantified for the transcript models using RSEM [33] and normalized within-samples to a fixed upper quartile for TCGA. Further details are available at the DCC data portal of TCGA. Gene-level data were restricted to genes expressed in at least 70% of samples. The complete dataset was normalized by the VOOM procedure [30], and it was split according to the TCGA-1 and 2 DNA methylation datasets.

### Molecular subtype

In all datasets the samples were classified into the three molecular glioma subtypes according to the revised WHO classification 2016 [36] using HM-450K data. The G-CIMP status was determined by unsupervised clustering (Ward’s algorithm with Euclidean distance) as previously reported [5]: the G-CIMP status served as approximation for the IDH mutation status, as this information was not available for all samples in any of the datasets. The IDH mutation status was available for 123/132 patients in EORTC 22033, 194/227 patients in AGlioma, 96/201 patients in TCGA-1, and 99/197 patients in TCGA-2. The 1p/19q codeletion status was assessed using the combined intensities for methylated and unmethylated signals and circular binary segmentation to detect copy number aberration (CNA) events as previously described [4]. Baseline characteristics are described in Table 1 for all four datasets.

**Table 1:** Clinical and molecular variables of the EORTC 22033 dataset and the external glioma datasets (WHO grade II and III) used for this study

Variable	Modality	EORTC 22033	TCGA-1	TCGA-2	AGlioma	*p* value
*N*		132	201	197	227	–
Sex^a^	M	74	114	111	72	0.6234
	F	58	87	86	42	
Age (year)^b^	Median	43	41	40	42	0.3683
	Min	20	17	14	23	
	Max	71	87	75	74	
Grade^a^	II	132	95	96	0	< 0.0001
	III	0	106	101	227	
Subtype^a,c^	IDHwt	12	37	36	50	0.0105
	IDHmt non-codel	80	108	107	97	
	IDHmt codel	40	56	54	80	

^a^ Chi-squared test

^b^ Kruskal and Wallis test

^c^ IDH status inferred from G-CIMP status; see Methods

### Estimation of tumor purity based on HM-450K

The tumor purity of each sample was estimated by a predictive model based on DNA methylation information using the purity estimation by the ABSOLUTE procedure available for the LGG TCGA dataset [7]. The split LGG TCGA datasets served as training and validation sets, respectively. The prediction of the purity of the samples was provided by a Sparse Partial Least Squares model (SPLS), where unmethylated (β-median < 0.2) CpG-probes located in intergenic regions were the predictors and ABSOLUTE purity estimation corresponded to the response (after arcsin-square-root transformation). The SPLS regression used the PLS-NIPALS algorithm (maximize covariance between variable of interest and predictors) with lasso regularization to reduce the number of dimensions and to limit multicollinearity [31]. The final model was used to predict purity (HMP index) for the four datasets (details in supplementary Fig. S1, Online Resource 1).

### Statistical analysis

Differential DNA methylation and differential gene expression between codeleted and non-codeleted IDHmt tumors were tested by moderated *t* test (R package limma) on *M* values [15] and normalized expression, respectively. Beta-values, equivalent to methylation fraction, were used to complement *M* values as a measure of effect size. The set of differentially methylated positions (DMPs) was established with a fold change cut-off for the Beta-value of 0.1 and for the fdr-adjusted *p* values of 0.1.

This analysis was performed for the four datasets and the list of candidate DMPs was given by the intersection of the four results. Comparison of DNA methylation patterns were investigated by a simultaneous analysis of several tables based on a principal component analysis algorithm called STATIS [29]. This method enables the analysis of the relationship among data tables with the same variables (CpG-probes) and combines them into a compromise matrix corresponding to the optimal agreement between the data. Briefly, STATIS can be separated into three steps: the interstructure, the analysis of the compromise and the intrastructure. The interstructure consists of the comparison of the table by RV-coefficient [44] and the construction of the compromise. The RV-coefficient measures the similarity between two tables: the values 0 and 1 correspond to unperfect and perfect matching between the two tables, respectively. A permutation test based on the first eigenvalue of the interstructure was developed to evaluate the strength of the relationship among datasets. The analysis of the compromise yields a reference system providing an optimal averaged representation of the columns (CpG-probes). The representation of the rows (samples) and columns (CpG-probes) of each table are obtained by projection onto the reference system (intrastructure). These analyses and graphics were performed using the R package ade4 [12].

Differential gene expression was considered as significant when the absolute fold change was superior or equal to log2 (1.2) and the fdr-adjusted *p* value inferior or equal to 0.1. Association between PFS and CpG-probes were assessed using the Cox proportional-hazards regression model and the log-likelihood ratio test (LRT). The additive models included CpG-probes, treatment or treatment and 1p/19q codeletion status as covariables, and contained the interaction term between CpG-probes and treatment. Time was taken from the date of surgery (not randomization as in the clinical trial) and an event (treatment failure/progression or death) after randomization to RT or TMZ. The predictive effect of markers was assessed by testing the interaction between treatment and CpG-probes.

The mean comparison between groups was performed by *t* test or by one-way anova. The Wald test with robust estimation of the covariance matrix was used when heteroskedasticity was suspected [52]. All analyses and graphical representations were performed using R-3.4.1 (https://www.R-project.org/).

## Results

### General description

Tumor DNA methylation profiles were available for analysis from 132 patients of 477 (28%) randomized in EORTC 22033 [6]. Classification of the samples into WHO subgroups based on the methylation data identified 40 (30.3%) G-CIMP 1p/19q codel (‘oligodendroglioma’), 80 (60.6%) G-CIMP 1p/19q non-codel (‘astrocytoma, IDHmt’), and 12 (9.1%) non-CIMP (‘astrocytoma, IDHwt’) tumors. The proportions of the molecular WHO subgroups were slightly different from those in the clinical trial subpopulation with available molecular classification (*n* = 318) [6]. The comparison of clinical baseline characteristics between this subset and the rest of the trial population is summarized in the supplementary Table S1 (Online Resource.1). There were less biopsy-only patients in our cohort, as expected, and the association of the 1p/19q codeletion status and PFS was different.

The IDH mutation status was not available for all tumor samples in any of the four datasets (available for 512/757; see ‘Molecular subtype’ in Methods), therefore, we used the G-CIMP status as proxy for the IDH mutation status. Where both were available, G-CIMP and *IDH1/2* mutation information agree almost perfectly, 100% for TCGA-1 and TCGA-2, 97.56% for EORTC 22033, and 97.42% for AGlioma. Throughout the manuscript we will refer to ‘IDH status’. Table 1 summarizes the proportions of the molecular WHO subtypes and basic clinical characteristics of the patients in the four datasets (EORTC 22033, TCGA1 and 2, AGlioma) used in the subsequent analyses.

### The DDR methylome of glioma

**Fig. 1:**
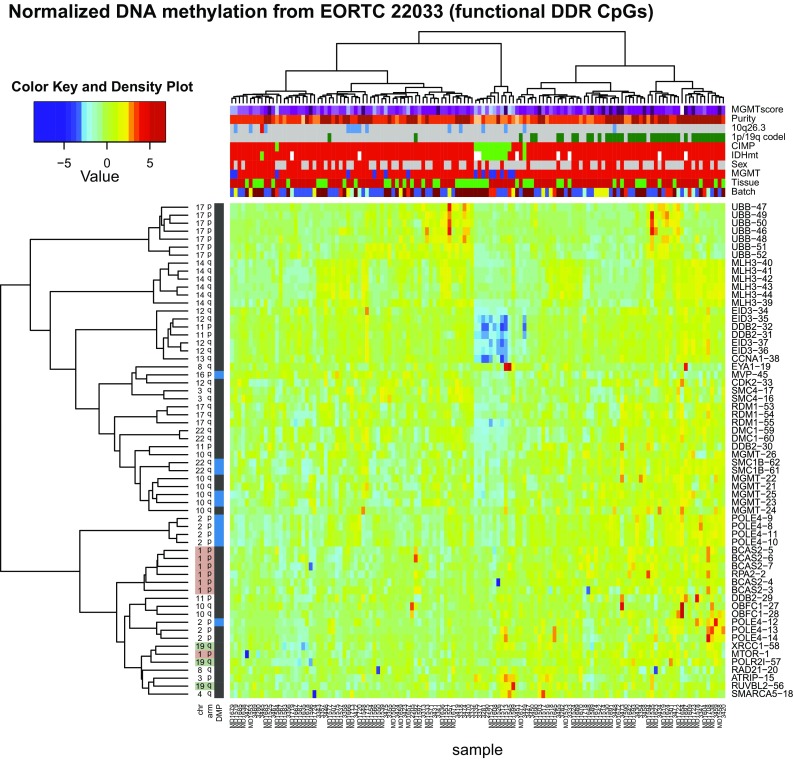
Heatmap representation of normalized DNA methylation of the 62 functional DDR CpGs in EORTC 22033. The dendrogram was established by hierarchical classification using Ward’s algorithm and Euclidean distance. Annotation of the 132 LGG samples comprises: *MGMT* methylation score (purple–blue color gradient, higher score more purple), purity estimation (HMP index, orange color gradient, more pure, more dark), copy number status for 10q26.3 region (grey, no change; red, amplification; blue, deletion), 1p/19q codeletion status (green, codeleted; grey, non-codeleted), CIMP status (green, non-CIMP; red, CIMP), *IDH1* and *IDH2* mutation status (green, wild type; red, IDH mutated; white, not determined), sex (red, female; grey, male), DNA methylation status of *MGMT* (MGMT-STP27, unmethylated, blue; methylated, red), tissue type (green, frozen; red, FFPE) and Batch (different colors). The 62 functional DDR CpGs are indicated on the right. The corresponding probe names are listed in supplementary Table S2 (Online Resource 2). The location on the chromosome (chr) arm, and the status as differential methylation position (DMP, blue; differentially methylated between codeleted and non-codeleted tumors) are indicated on the left

We aimed at identifying DDR genes down-regulated by aberrant promoter methylation that could either serve as biomarkers for outcome prediction or inform on inherent DNA repair vulnerabilities amenable to treatment. To this end the split TCGA LGG datasets (which includes both grade II and III glioma) were used for the selection of functionally methylated CpGs located in the promoters of DDR genes as outlined in supplementary Fig. S2 (Online Resource 1). Functional methylation was defined under the postulate that DNA methylation of the promoter down-regulates expression. The HM-450K chip annotates 3749 CpGs as located in the promoter regions of 410 DDR genes. Thereof 62 CpGs satisfied the criteria of functional methylation in both datasets, identifying 24 DDR genes (supplementary Table S2, Online Resource 2). The functionally methylated probes are displayed for the EORTC 22033 dataset in a heatmap in Fig. 1, annotated with key clinical and molecular information. The heatmap visualizes the high concordance of CpG methylation within the individual genes. This subset of methylation probes largely separated the LGG according to the molecular subtypes.

For the subsequent data analyses, we only considered IDHmt LGG. IDH wild-type (IDHwt) astrocytoma were rare in our cohort (*n* = 12); furthermore, they are considered a heterogeneous group that may belong to other tumor entities [43].

First, we aimed at demonstrating the robustness of the methylation patterns of interest across the four datasets as a prerequisite for the validity of the following analyses. The two TCGA datasets (TCGA1 and 2) were highly similar in regard to the functional methylation of the selected 62 functional CpGs in DDR genes, as determined by comparing their Pearson correlation between gene methylation and gene expression [RV-coefficient = 0.92 (max. achievable value is 1), *p* value = 0.001 for 999 permutations; supplementary Fig. S3, Online Resource 1].

**Fig. 2:**
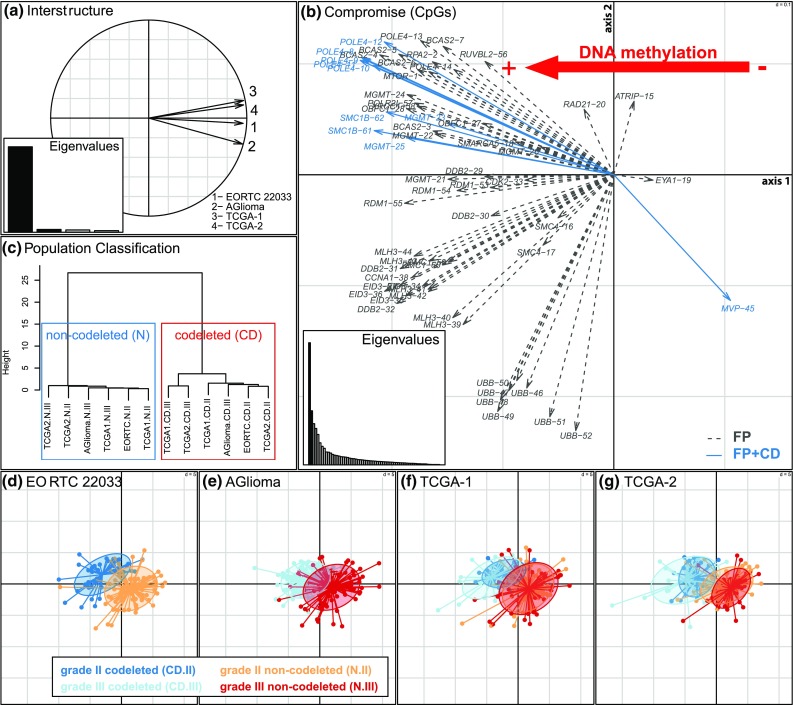
Similarity of functional DNA methylation patterns of IDHmt LGG among the four datasets. **a** The four DNA methylation datasets, using the common 62 functional DDR CpGs, were analyzed simultaneously by the STATIS procedure to determine similarity. First, the global comparison of the four datasets is provided by the *interstructure*, based on the pairwise comparisons (RV coefficients, all > 0.8). Each dataset is represented by an arrow and the small angles between arrows indicate high similarity between datasets (global permutation test, simulation *p* value < 0.001 for 999 permutations). **b** The *compromise* (PCA-like) analysis of the functional DDR CpGs across the datasets provides an optimized average representation. It gives a general view of the correlations between the functional CpGs (see supplementary Table S2, Online Resource 2, for the list of corresponding CpG probes). The gradient of CpG methylation is indicated with the red arrow. The CpGs detected as significantly differentially methylated between codeleted and non-codeleted groups are represented by blue arrows. The representation of the patients on the two first axes of the compromise analysis is given for each dataset (**d**, **e**, **f**, **g**). The first axes mainly separates the samples according to the codeletion status, as visualized by the inertia ellipse [CD-II, grade II codeleted (blue); CD-III, grade III codeleted (light blue); N-II, grade II non-codeleted (orange); N-III, grade III non-codeleted (red)]. **c** In line, the clustering of the molecular subgroups of the four datasets separates the codeleted (blue) from the non-codeleted (red) samples (Ward’s algorithm and Euclidean distance derived from STATIS coordinates and is visualized in a dendrogram)

Next, a common DNA methylation pattern among the four datasets was confirmed for the 62 CpGs (Fig. 2a, STATIS interstructure, global permutation test, simulation *p* value < 0.001 for 999 permutations). In concordance, pairwise comparison of the methylation patterns in the four datasets (TCGA-1 and 2, AGlioma and EORTC 22033) showed high similarity with all RV coefficients > 0.8 (STATIS analysis) (Fig. 2a).

An overview of the correlations between CpGs of all four datasets is visualized in Fig. 2b showing the consensus representation of the functional CpG probes (first two axes). Genes featuring multiple functionally methylated CpGs in their promoter, such as *UBB*, *MLH3*, *MGMT*, *POLE4* and *BCAS2,* highlight the high concordance of methylation among their CpGs, confirming the robustness of DNA methylation patterns at the gene scale and across datasets (Fig. 2b). This consensus gene organization indicates a strong DNA methylation gradient implying a higher extent of methylation in IDHmt codeleted tumors, as compared to IDHmt non-codeleted tumors (Fig. 2b). Accordingly, differences between the IDHmt codeleted and non-codeleted subpopulations were observed for all four datasets (Fig. 2d–g). The separation of the IDHmt codeleted and non-codeleted subgroups across all four datasets is also demonstrated by cluster analysis (Fig. 2c).

**Fig. 3:**
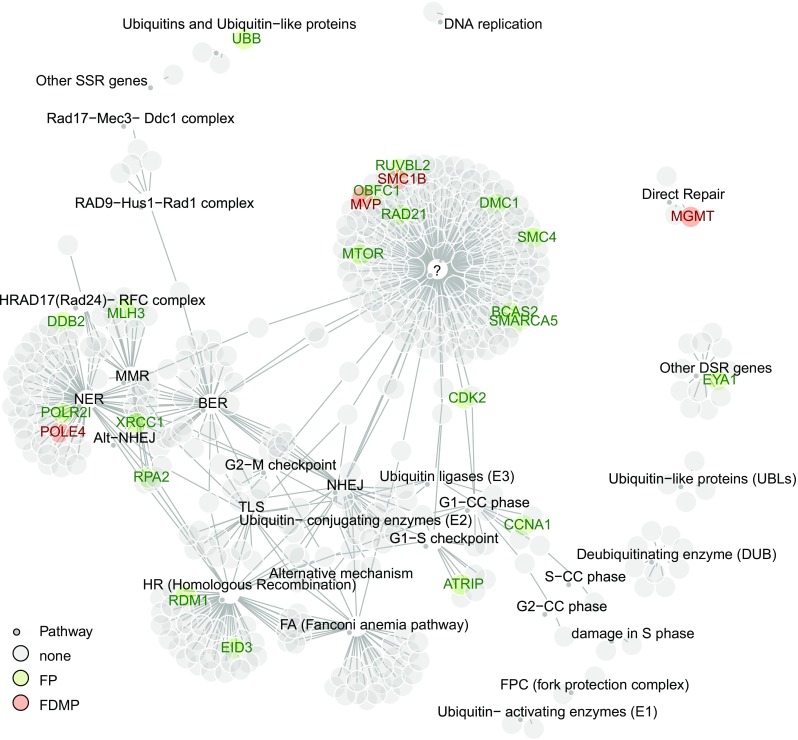
DDR networks based on pathways as defined in Pearl et al. [40]. DDR genes identified as functionally methylated are indicated and marked in green (FP). Functional DDR genes that are differentially methylated between IDHmt codel and IDHmt non-codel LGG are marked in red (FDMP)

The functional probeset included *MGMT* that is of known relevance for DNA repair associated with TMZ treatment-induced lesions, and the putative mismatch repair (MMR) gene *MLH3*. Other functionally methylated DDR genes identified have been associated with nuclear excision repair (NER), e.g. *POLE4*, or homologous recombination (HR), e.g. *RDM1*. The gene encoding UBB was selected with a large number of functionally methylated CpGs. It is involved in regulation of chromatin structure and protein degradation affecting many pathways. The identified 24 functionally methylated genes are visualized in the context of the DDR network in Fig. 3. No enrichment for specific repair pathways was observed.

### DDR genes associated with 1p/19q codeletion in LGGs

The molecular subtypes have been associated with differential benefit from TMZ vs RT; e.g. patients with non-codeleted IDHmt LGG did not benefit as much from TMZ as from RT [6], suggesting differences in the proficiency of some DNA repair systems. Therefore, we assessed differential DNA methylation (DM) between codeleted and non-codeleted IDH-mutant samples for CpGs located in the promoters of DDR genes. This identified 36 differentially methylated positions (DMP) corresponding to 19 genes (supplementary Fig. S4, Online Resource 1; supplementary Table S2, Online Resource 2); one-third of them belong to 4 functionally methylated DDR genes (10/36 CpGs: 2 CpGs for *MGMT*, 5 CpGs for *POLE4*, 1 CpG for *MVP*, and 2 CpGs for *SMC1B*; Fig. 3, supplementary Table S2, Online Resource 2).

Gene expression analyses in the TCGA-1 and 2 datasets identified 74 of 410 DDR genes as differentially expressed (DE): 39 genes down-regulated in the codeleted group, and 35 up-regulated (supplementary Table S3, Online Resource 3 and supplementary Fig. S5, Online Resource 1). The top up-regulated DDR gene in IDHmt codeleted tumors was *hTERT,* whose activating promoter mutations are known to be correlated with overexpression and 1p/19q codeletion (supplementary Table S3, Online Resource 3) [2]. Three downregulated DDR genes (*MGMT, POLE4* and *SMC1B*) were differentially methylated, 22 genes are located on either chromosome 1p or 19q, and downregulation may be attributed to gene dosage (loss of one allele). No significant enrichment of a given DDR pathway was observed using gene set enrichment analysis (GSEA) (based on mean-rank gene set test, not shown).

### Predictive markers for progression-free survival in IDHmt LGG of EORTC 22033

The EORTC 22033 dataset allows for correlation of gene methylation with outcome and influence of treatment modality. In 120 IDHmt patient samples of EORTC 22033, we tested the 62 functionally methylated CpGs identified in 24 DDR genes for their association with PFS depending on treatment (supplementary Table S4, Online Resource 4). This was investigated by testing for significance of the treatment–biomarker interaction term in a Cox proportional-hazards survival model. The analysis yielded 7 CpGs located in the promoters of 4 DDR genes: 3 of 6 functional CpGs in *MLH3*, 2 of 6 in *MGMT*, and 1 of 2 in *SMC4* and 1 of 1 in *RAD21*, respectively. The expected survival by treatment arm, in function of the continuous CpG methylation values, is visualized in the supplementary Fig. S6 (Online Resource 1). The results suggested that the two leading CpGs for *MGMT* are prognostic for PFS in the TMZ arm only, while the top *MLH3* CpGs and those from *SMC4 and RAD21* are prognostic in the radiation arm only. The same 7 CpGs were retained when the model included the codeletion status as an adjustment factor (supplementary Table S4, Online Resource 4).

**Fig. 4:**
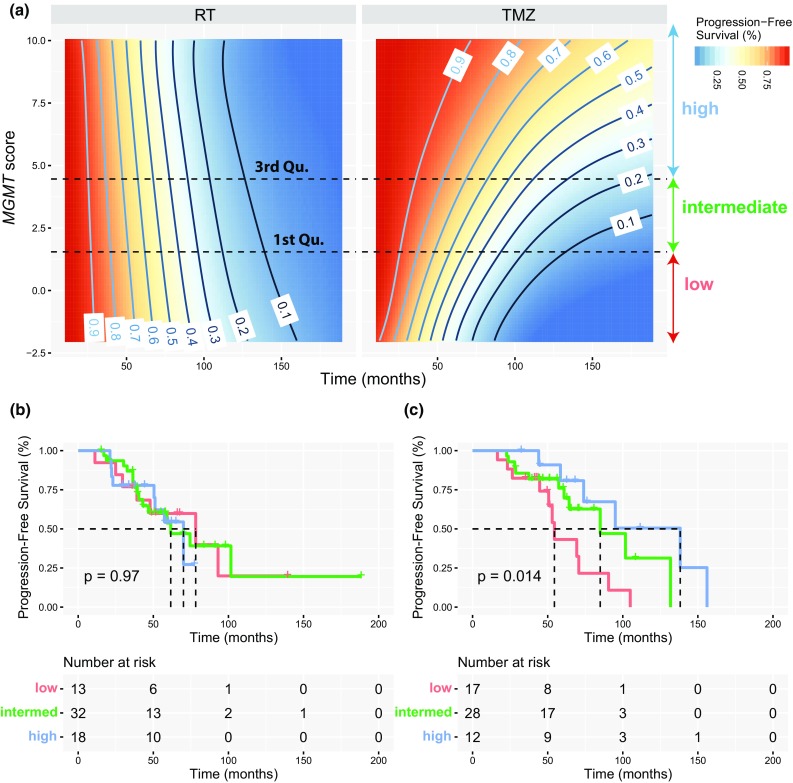
Progression-free survival in function of the *MGMT* methylation score. **a** The simulation of progression-free survival (%, PFS) is based on the Cox proportional-hazards model and PFS (%), and is shown in function of time (month) and the *MGMT* methylation score for patients randomized to treatment with radiotherapy (RT) or temozolomide (TMZ). The graphics illustrate a three-dimensional Kaplan–Meier plot, where the *MGMT* methylation score and time are explanatory variables and the colors of the gradient complemented by contour lines indicate progression-free survival (%). As examples, two cut-offs (first and third quartiles of *MGMT* score distribution) were used to define three groups of methylation (low, intermediate, high) for patients from EORTC 22033. The two cut-offs are indicated by dashed lines in **a**. Kaplan–Meier plots illustrate the association between a high *MGMT* score and PFS in the treatment arms, RT (**b**) and TMZ (**c**). The *p* values are given by log-rank test. The KMs in **b** and **c** confirm the significant interaction between TMZ and the *MGMT* score (Table 2)

The two CpGs selected for *MGMT* were identical to those we previously selected for the logistic regression model to calculate the *MGMT* methylation score used by the MGMT-STP27 classifier [4, 5]. The interaction term with treatment was also significantly different from zero for this score [Table 2, HR = 0.755, 95% CI (0.5786, 0.9772), *p* value = 0.033, Fig. 4a], independently whether the codeletion status was added to the model (Table 2). As example, Kaplan–Meier representations are given for two cut-offs (first and third quartiles of *MGMT* score distribution) that illustrate the interaction effect between high *MGMT* methylation and treatment with TMZ in EORTC 22033 (Fig. 4b, c). Of note, the codeletion status was not significantly associated with PFS among IDHmt patients in a univariable model (*p* = 0.16, log rank test).

**Table 2:** Multi-variable models for progression-free survival (PFS)

Variable	Model	Variable	Coefficient	HR	SE	*z* value	*p* value
*MGMT* methylation score (raw)	Model 1	*MGMT*	0.0502	1.0515	0.0912	0.5502	0.5822
		TRT (TMZ)	− 0.0797	0.9234	0.2770	− 0.2876	0.7736
		*MGMT* × TRT	− 0.2851	0.7519	0.1337	− 2.1324	**0.0330**
	Model 2	CODEL (CD)	− 0.1988	0.8197	0.3281	− 0.6060	0.5445
		*MGMT*	0.0667	1.0689	0.0948	0.7030	0.4821
		TRT (TMZ)	− 0.0462	0.9548	0.2821	− 0.1639	0.8698
		*MGMT* × TRT	− 0.2807	0.7553	0.1342	− 2.0910	**0.0365**
*MGMT* methylation score after removing effect of purity (HMP index)	Model 1	*MGMT*	0.0715	1.0741	0.1028	0.6960	0.4864
		TRT (TMZ)	− 0.1778	0.8371	0.2728	− 0.6519	0.5145
		*MGMT* × TRT	− 0.2942	0.7451	0.1407	− 2.0910	**0.0365**
	Model 2	CODEL (CD)	− 0.2464	0.7816	0.3168	− 0.7778	0.4367
		*MGMT*	0.0895	1.0936	0.1044	0.8570	0.3915
		TRT (TMZ)	− 0.1299	0.8782	0.2790	− 0.4656	0.6415
		*MGMT* × TRT	− 0.2910	0.7475	0.1404	− 2.0720	**0.0383**

Values in bold are statistically significant

*TRT* treatment

**Fig. 5:**
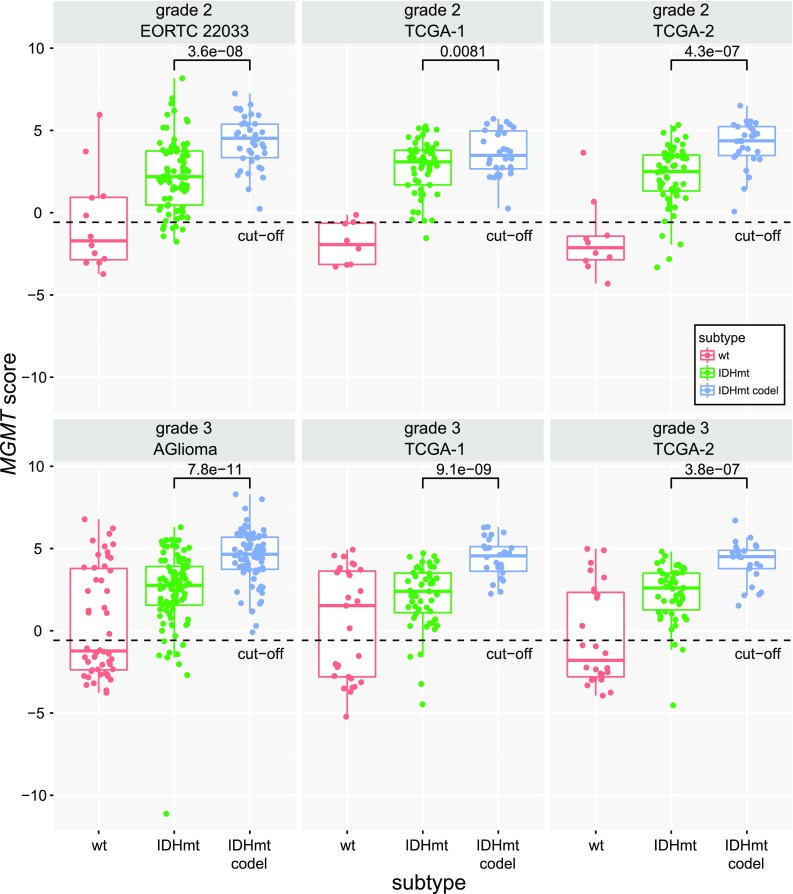
*MGMT* score in function of the molecular subtype in LGG. The *MGMT* methylation scores for the three distinct molecular subtypes (wt, CIMP-/IDHwt;  IDHmt, CIMP+ non-codel;  IDHmt codel, CIMP+ codel) separated by WHO tumor grade, are shown for the four datasets (EORTC 22033, TCGA1 & 2, AGlioma). The *p* values of the comparison between codeleted and non-codeleted samples are based on the Welch’s *t* test for each dataset. The dashed line indicates the MGMT-STP27 cut-off [logit(0.358)]

The *MGMT* methylation score was significantly higher in the codeleted than in the non-codel IDHmt glioma in all four datasets (Fig. 5). A scatter plot of the *MGMT* methylation score and *MGMT* expression visualizing the negative correlation is displayed for the three molecular LGG subtypes of the TCGA dataset, for which RNA-seq expression data are available (supplementary Fig. S7, Online Resource 1).

It has been reported that tumor purity, “contaminated” by microglia, non-tumoral brain tissue, and tumor infiltrating cells, differs between histologic subtypes of LGG [1] and may therefore be a confounding factor for estimating subtype dependent differences of DNA methylation. In order to exclude that the higher *MGMT* methylation score in the codeleted tumors is driven by higher purity, we estimated sample purity based on the methylation data (see methods). Significant differences between the codeleted and non-codeleted IDHmt tumors (*p* value < 0.01, *t* test, supplementary Fig. S8, Online Resource 1) were observed in all four datasets (EORTC 22033, TCGA1 and 2, AGlioma). These results confirm that codeleted IDH-mutant tumors appear more pure. A significant but only weak association between the *MGMT* score and tumor sample purity was detected for EORTC 22033 (*p* value = 0.0013, Wald test based on robust estimation). The purity index explained 7.1% of the variance. Adjusting the *MGMT* score for the purity effect using a linear model had only a minor effect on the association of the *MGMT* score with the molecular subtype (supplementary Fig. S9, Online Resource 1) and led only to minor modifications in the Cox proportional-hazards models and the significance tests in EORTC 22033 [Table 2, HR = 0.7451, 95% CI (0.5655, 0.9817), *p* value = 0.037]. Similarly, a weak association between the *MGMT* score and purity was observed for the TCGA-1 (*p* value < 0.0001, *R*^2^ = 7.1%) and TCGA-2 (*p* value < 0.0001, *R*^2^ = 6.7%) datasets, whereas the explained variance was higher in the AGlioma dataset (*R*^2^ = 22.6%, *p* value < 0.0001).

## Discussion

Here we set out to investigate whether epigenetic inactivation of DDR genes affected the patients’ benefit (PFS, time lapse from initial surgery to treatment failure upon randomization to RT or TMZ therapy) from genotoxic treatment with either RT or TMZ in EORTC 22033. Furthermore, we aimed at uncovering potential DDR pathway vulnerabilities as potential drug targets. Through multidimensional analyses of the molecular LGG dataset of TCGA, we identified 24 candidate DDR genes that are functionally downregulated by aberrant promoter methylation. Importantly, promoter methylation of four of these DDR genes was predictive for benefit (PFS) from either alkylating agent chemotherapy (TMZ) or RT in EORTC 22033.

The most prominent functionally methylated gene was *MGMT* that is known as predictive factor for benefit from TMZ in GBM [22]. Most IDHmt LGG have a methylated *MGMT* gene promoter [4–6, 18]. In line, in this study all IDHmt 1p/19q codel LGG, and almost 90% of the IDHmt non-codel glioma were *MGMT* methylated [6]. Due to this nested relationship, the mechanistic impact of *MGMT* promoter methylation on treatment-related outcome remains unclear. The two *MGMT* CpG probes identified in this study as predictive, are also the ones selected in the MGMT-STP27 classifier to calculate the score, and both are predictive for benefit from treatment with alkylating agents in GBM [5]. Most importantly, in this study an increased *MGMT* methylation score (~ extent of *MGMT* methylation) was predictive for benefit under TMZ treatment, but not under RT. The *MGMT* score was significantly higher in codeleted vs non-codeleted IDHmt tumors. This is in agreement with the clinical observation of a worse PFS of IDHmt non-codeleted patients when treated with TMZ, while there was no difference when treated with radiotherapy [6].

There are some notable differences of *MGMT* methylation in LGG compared to GBM. In IDHmt LGG the *MGMT* methylation score summarizes methylation of two *MGMT* alleles, while GBM harbor frequent loss of one allele (> 80%; CHR 10q26) [4], and the methylation of the retained allele informs on inactivation of the *MGMT* gene. Thus, in presence of two alleles, the detection of methylation may indicate methylation of both alleles, or complete methylation of only one allele, leaving the gene on the remaining allele functional. Thus, a high *MGMT* methylation score increases the probability of inactivating both *MGMT* alleles.

The relevance, and mechanistic implications of epigenetic silencing, of the other three genes identified as predictive for benefit from RT, are less clear. Methylation of three CpGs in the *MLH3* promoter appear predictive for benefit from RT. MLH3 is usually associated with MMR, although it seems to play only a minor role in this process, and may not actually be required [9, 26]. Germline mutations in *MLH3* have been associated with the Lynch syndrome, although not fulfilling the Amsterdam I criteria, and with an unclear clinical role [42]. Frequent *MLH3* methylation in IDHmt LGG has been reported previously [32]; MLH3 may be involved in other cancer-relevant processes [9].

For the other two genes only one CpG each was associated with outcome and treatment. RAD21 is involved in DSB repair, and haploinsufficiency has been reported to enhance radiosensitivity in a mouse model [51], in line with a potential predictive value for RT. SMC4 is part of the condensin complexes I and II that are essential for chromosome assembly and segregation, and is involved DSB repair [34]. Genes encoding members of the cohesion complex are targeted by deletion or mutation in 16% of LGG/GBM, suggestive of a glioma genesis relevant role of the pathway [11].

The search for biomarkers identifying potential vulnerabilities in repair pathways yielded a number of candidates. Twenty-four functionally methylated DDR genes were confirmed in three independent LGG datasets, and some may open novel treatment options. Most interestingly, recent encouraging results from clinical trials have raised the interest in targeting DNA repair vulnerabilities in cancer. Responses to the PARP inhibitor olaparib have been observed in metastatic prostate cancer with defects of repair genes (mutations, deletions) [37]. In breast cancer inactivation of *BRCA1* and/or *2* by promoter methylation is currently considered for treatment with the same PARP inhibitor in a trial (clinicaltrial.gov, NCT03205761). Veliparib is currently undergoing phase 3 evaluation in GBM in a randomized Alliance trial (NCT02152982). Along the same lines, PARP inhibitors may be useful for LGG patients with promoter methylation of *MLH3*, or *XRCC1* that when defective have been reported to render cells sensitive to PARP inhibition, and are therefore discussed as being potentially amenable to PARP inhibitor treatments [37, 38].

Of interest, *XRCC1* is located on CHR 19q, hence haploinsufficient in the IDHmt codeleted tumors. Consequently, detection of promoter methylation informs on the silencing of the remaining allele. Furthermore, based on predictions for druggability of DDR genes (see recent reviews by Pearl et al. [41] and Stover et al. [47]), five of our functionally methylated DDR genes (*RPA2 SMARCA5, SMC4, XRCC1, mTOR*) may warrant further investigations. Targeting of mTOR is currently under investigation in a trial for LGG with or without TMZ treatment (NCT02023905).

The current efforts to promote the HM-450K methylation platform (or the more recent version, EPIC) as a diagnostic tool for classification of brain tumors [10, 17], will make the here identified markers potentially evaluable in routine diagnostics and may allow validation of the presented results. This diagnostic tool integrates the *MGMT* methylation score as part of the MGMT-STP27 classifier [10]. However, most quantitative assays for *MGMT* methylation analysis, such as methylation-specific pyrosequencing should be amenable to determine the extent of methylation. We previously showed good concordance between the HM-450K based MGMT-STP27 classifier and pyrosequencing using the respective cut-offs for glioblastoma [5].

We have focused our investigations to epigenetically silenced DDR genes, since promoter methylation seems to be quite stable in tumors and may not easily change under treatment [19, 28]. While mutations in DDR genes are exceedingly rare in chemo-naive LGG, with the exception of *TERTp* and *ATRX* [48], there are other mechanisms attenuating relevant DNA repair systems. Of particular interest for lower grade glioma is the recently described inhibitory effect of the oncometabolite 2-hydroxy glutarate (2HG) on the α-ketoglutarate-dependent ALKBH repair enzymes that are involved in direct DNA repair, including TMZ induced lesions [24, 49]. 2HG, produced by the neomorphic function of the IDH1 and 2 mutants is accumulated to high concentrations in the respective tumors [13] and may confer sensitivity to alkylating agent chemotherapy and protracted natural history.

In conclusion, our analyses suggest that a high *MGMT* methylation score predicts PFS in TMZ-treated patients with IDHmt tumors regardless of the 1p/19q status. This information may guide clinical decision-making for individual patients, and in particular when considering deferring radiotherapy in patients with a better prognosis aiming at avoiding or delaying potential neurocognitive toxicity.

The limitation of the present retrospective study is the low statistical power and the current lack of a comparable validation set. Data for OS are not available yet. However, the development of predictive markers to allow treatment de-escalation are important in the future, personalizing treatment strategy (single vs combined modality, choice of modality, novel targets) needs to be risk-adapted and is of particular importance in good-prognosis disease.

## Electronic supplementary material

Below is the link to the electronic supplementary material.

Supplementary material 1 (PDF 962 kb)

Supplementary material 2 (XLSX 31 kb)

Supplementary material 3 (XLSX 29 kb)

Supplementary material 4 (XLSX 41 kb)

## References

[1] Aran D, Sirota M, Butte AJ (2015). Systematic pan-cancer analysis of tumour purity. Nat Commun.

[2] Arita H, Narita Y, Fukushima S, Tateishi K, Matsushita Y, Yoshida A (2013). Upregulating mutations in the TERT promoter commonly occur in adult malignant gliomas and are strongly associated with total 1p19q loss. Acta Neuropathol.

[3] Aryee MJ, Jaffe AE, Corrada-Bravo H, Ladd-Acosta C, Feinberg AP, Hansen KD (2014). Minfi: a flexible and comprehensive Bioconductor package for the analysis of Infinium DNA methylation microarrays. Bioinformatics.

[4] Bady P, Delorenzi M, Hegi ME (2016). Sensitivity analysis of the MGMT-STP27 model and impact of genetic and epigenetic context to predict the MGMT methylation status in gliomas and other tumors. J Mol Diagn.

[5] Bady P, Sciuscio D, Diserens AC, Bloch J, van den Bent MJ, Marosi C (2012). MGMT methylation analysis of glioblastoma on the Infinium methylation BeadChip identifies two distinct CpG regions associated with gene silencing and outcome, yielding a prediction model for comparisons across datasets, tumor grades, and CIMP-status. Acta Neuropathol.

[6] Baumert BG, Hegi ME, van den Bent MJ, von Deimling A, Gorlia T, Hoang-Xuan K (2016). Temozolomide chemotherapy versus radiotherapy in high-risk low-grade glioma (EORTC 22033-26033): a randomised, open-label, phase 3 intergroup study. Lancet Oncol.

[7] Brat DJ, Verhaak RG, Aldape KD, Yung WK, Salama SR, Cooper LA (2015). Comprehensive, integrative genomic analysis of diffuse lower-grade gliomas. N Engl J Med.

[8] Buckner JC, Shaw EG, Pugh SL, Chakravarti A, Gilbert MR, Barger GR (2016). Radiation plus procarbazine, CCNU, and vincristine in low-grade glioma. N Engl J Med.

[9] Cannavo E, Marra G, Sabates-Bellver J, Menigatti M, Lipkin SM, Fischer F (2005). Expression of the MutL homologue hMLH3 in human cells and its role in DNA mismatch repair. Cancer Res.

[10] Capper D, Jones DTW, Sill M, Hovestadt V, Schrimpf D, Sturm D et al (in press) DNA methylation-based classification of human central nervous system tumours. Nature 10.1038/nature26000 PMC6093218 29539639

[11] Ceccarelli M, Barthel FP, Malta TM, Sabedot TS, Salama SR, Murray BA (2016). Molecular profiling reveals biologically discrete subsets and pathways of progression in diffuse glioma. Cell.

[12] Chessel D, Dufour AB, Thioulouse J (2004). The ade4 package-I-one-table methods. R News.

[13] Dang L, White DW, Gross S, Bennett BD, Bittinger MA, Driggers EM (2009). Cancer-associated IDH1 mutations produce 2-hydroxyglutarate. Nature.

[14] Douw L, Klein M, Fagel SS, van den Heuvel J, Taphoorn MJ, Aaronson NK (2009). Cognitive and radiological effects of radiotherapy in patients with low-grade glioma: long-term follow-up. Lancet Neurol.

[15] Du P, Zhang X, Huang CC, Jafari N, Kibbe WA, Hou L (2010). Comparison of Beta-value and *M* value methods for quantifying methylation levels by microarray analysis. BMC Bioinf.

[16] Duncan CG, Barwick BG, Jin G, Rago C, Kapoor-Vazirani P, Powell DR (2012). A heterozygous IDH1(R132H/WT) mutation induces genome-wide alterations in DNA methylation. Genome Res.

[17] Eckel-Passow J, Decker P, Hughes E, Kollmeyer T, Kosel M, Burgenske D (2017). PATH-47. Clinical sensitivity and specificity of illumina methylation array for classifying adult gliomas into WHO groups. Neurooncology.

[18] Eckel-Passow JE, Lachance DH, Molinaro AM, Walsh KM, Decker PA, Sicotte H (2015). Glioma groups based on 1p/19q, IDH, and TERT promoter mutations in tumors. N Engl J Med.

[19] Felsberg J, Thon N, Eigenbrod S, Hentschel B, Sabel MC, Westphal M (2011). Promoter methylation and expression of MGMT and the DNA mismatch repair genes MLH1, MSH2, MSH6, and PMS2 in paired primary and recurrent glioblastomas. Int J Cancer.

[20] Fortin JP, Labbe A, Lemire M, Zanke BW, Hudson TJ, Fertig EJ (2014). Functional normalization of 450k methylation array data improves replication in large cancer studies. Genome Biol.

[21] Gusyatiner O, Hegi ME (2017) Glioma epigenetics: from subclassification to novel treatment options. Semin Cancer Biol. 10.1016/j.semcancer.2017.11.010 10.1016/j.semcancer.2017.11.010 29170066

[22] Hegi ME, Diserens AC, Gorlia T, Hamou MF, de Tribolet N, Weller M (2005). MGMT gene silencing and benefit from temozolomide in glioblastoma. N Engl J Med.

[23] Jenkins RB, Blair H, Ballman KV, Giannini C, Arusell RM, Law M (2006). A t(1;19)(q10;p10) mediates the combined deletions of 1p and 19q and predicts a better prognosis of patients with oligodendroglioma. Cancer Res.

[24] Johannessen TC, Prestegarden L, Grudic A, Hegi ME, Tysnes BB, Bjerkvig R (2013). The DNA repair protein ALKBH2 mediates temozolomide resistance in human glioblastoma cells. Neuro Oncol.

[25] Johnson WE, Li C, Rabinovic A (2007). Adjusting batch effects in microarray expression data using empirical Bayes methods. Biostatistics.

[26] Korhonen MK, Raevaara TE, Lohi H, Nystrom M (2007). Conditional nuclear localization of hMLH3 suggests a minor activity in mismatch repair and supports its role as a low-risk gene in HNPCC. Oncol Rep.

[27] Kurscheid S, Bady P, Sciuscio D, Samarzija I, Shay T, Vassallo I (2015). Chromosome 7 gain and DNA hypermethylation at the HOXA10 locus are associated with expression of a stem cell related HOX-signature in glioblastoma. Genome Biol.

[28] Laffaire J, Everhard S, Idbaih A, Criniere E, Marie Y, de Reynies A (2010). Methylation profiling identifies 2 groups of gliomas according to their tumorigenesis. Neuro Oncol.

[29] Lavit C, Escoufier Y, Sabatier R, Traissac P (1994). The act (statis method). Comput Stat Data Anal.

[30] Law CW, Chen Y, Shi W, Smyth GK (2014). voom: precision weights unlock linear model analysis tools for RNA-seq read counts. Genome Biol.

[31] Le Cao KA, Rossouw D, Robert-Granie C, Besse P (2008). A sparse PLS for variable selection when integrating omics data. Stat Appl Genet Mol Biol.

[32] Lhotska H, Zemanova Z, Cechova H, Ransdorfova S, Lizcova L, Kramar F (2015). Genetic and epigenetic characterization of low-grade gliomas reveals frequent methylation of the MLH3 gene. Genes Chromosom Cancer.

[33] Li B, Dewey CN (2011). RSEM: accurate transcript quantification from RNA-Seq data with or without a reference genome. BMC Bioinf.

[34] Losada A, Hirano T (2005). Dynamic molecular linkers of the genome: the first decade of SMC proteins. Genes Dev.

[35] Louis DN, Ohgaki H, Wiestler OD, Cavenee WK, Bosman FT, Jaffe ES, R LS, Ohgaki H (2016). WHO classification of tumours of the central nervous system. World Health Organization classification of tumours.

[36] Louis DN, Perry A, Reifenberger G, von Deimling A, Figarella-Branger D, Cavenee WK (2016). The 2016 World Health Organization Classification of tumors of the central nervous system: a summary. Acta Neuropathol.

[37] Mateo J, Carreira S, Sandhu S, Miranda S, Mossop H, Perez-Lopez R (2015). DNA-repair defects and olaparib in metastatic prostate cancer. N Engl J Med.

[38] Michels J, Vitale I, Saparbaev M, Castedo M, Kroemer G (2014). Predictive biomarkers for cancer therapy with PARP inhibitors. Oncogene.

[39] Noushmehr H, Weisenberger DJ, Diefes K, Phillips HS, Pujara K, Berman BP (2010). Identification of a CpG island methylator phenotype that defines a distinct subgroup of glioma. Cancer Cell.

[40] Pearl J (1998). Graphs, causality, and structural equation models. Sociol Method Res.

[41] Pearl LH, Schierz AC, Ward SE, Al-Lazikani B, Pearl FM (2015). Therapeutic opportunities within the DNA damage response. Nat Rev Cancer.

[42] Peltomaki P (2005). Lynch syndrome genes. Fam Cancer.

[43] Reuss DE, Kratz A, Sahm F, Capper D, Schrimpf D, Koelsche C (2015). Adult IDH wild type astrocytomas biologically and clinically resolve into other tumor entities. Acta Neuropathol.

[44] Robert P, Escoufier Y (1976). A unifying tool for linear multivariate statistical methods: the RV-coefficient. Appl Stat.

[45] Ruda R, Soffietti R (2017). Controversies in management of low-grade gliomas in light of new data from clinical trials. Neuro Oncol.

[46] Schiff D (2016). Molecular profiling optimizes the treatment of low-grade glioma. Neuro Oncol.

[47] Stover EH, Konstantinopoulos PA, Matulonis UA, Swisher EM (2016). Biomarkers of response and resistance to DNA repair targeted therapies. Clin Cancer Res.

[48] Suzuki H, Aoki K, Chiba K, Sato Y, Shiozawa Y, Shiraishi Y (2015). Mutational landscape and clonal architecture in grade II and III gliomas. Nat Genet.

[49] Wang P, Wu J, Ma S, Zhang L, Yao J, Hoadley KA (2015). Oncometabolite D-2-Hydroxyglutarate inhibits ALKBH DNA repair enzymes and sensitizes IDH mutant cells to alkylating agents. Cell Rep.

[50] Wiestler B, Capper D, Sill M, Jones DT, Hovestadt V, Sturm D (2014). Integrated DNA methylation and copy-number profiling identify three clinically and biologically relevant groups of anaplastic glioma. Acta Neuropathol.

[51] Xu H, Balakrishnan K, Malaterre J, Beasley M, Yan Y, Essers J (2010). Rad21-cohesin haploinsufficiency impedes DNA repair and enhances gastrointestinal radiosensitivity in mice. PLoS One.

[52] Zeileis A (2004). Econometric computing with hc and hac covariance matrix estimators. J Stat Software.

