# Supplementary material for: The DNA methylome of DDR genes and benefit from RT or TMZ in IDH mutant low-grade glioma treated in EORTC 22033
Source: Acta Neuropathol. 2018 Jan 24;135(4):601–15. doi: 10.1007/s00401-018-1810-6 (PMC5978935; doi:10.1007/s00401-018-1810-6)
Supplement: Supplementary file 1 — Supplementary material 1 (PDF 962 kb) [file 401_2018_1810_MOESM1_ESM.pdf]

The DNA methylome of DDR genes and benefit from RT or TMZ in IDH mutant low grade glioma treated in EORTC 22033

Authors: Pierre Bady[1-4], Sebastian Kurscheid[1,2,4,5], Mauro Delorenzi[4,6,7], Thierry Gorlia[8], Martin van den Bent[9], Khê Hoang-Xuan[10], Élodie Vauleon[11], Anja Gijtenbeek[12], Roelien Enting[13], Brian Thiessen[14], Olivier Chinot[15], Frédéric Dhermain[16], Alba A. Brandes[17], Jaap C. Reijneveld[18], Christine Marosi[19], Martin JB. Taphoorn[20], Wolfgang Wick[21], Andreas von Deimling[22], Pim French[9], Roger Stupp[2,23], Brigitta G. Baumert [24,25], Monika E. Hegi[1,2]

[1]Neuroscience Research Center, Lausanne University Hospital, Lausanne, Switzerland;
[2]Division of Neurosurgery, Lausanne University Hospital, Lausanne, Switzerland;
[3]Department of Education and Research, Lausanne University Hospital, Lausanne, Switzerland
[4]Bioinformatics Core Facility, Swiss Institute for Bioinformatics SIB, Lausanne, Switzerland
[5]Department of Genome Science, The Australian National University, Canberra, Australia
[6]Department of Oncology, Lausanne University Hospital, Lausanne, Switzerland
[7]Ludwig Center for Cancer Research, University of Lausanne, Switzerland
[8]EORTC Headquarter, Brussels, Belgium
[9]Erasmus MC Cancer Institute University Medical Center, Rotterdam, The Netherlands
[10]APHP Pitié-Salpétrière, Sorbonne Universités, UPMC, UMR S 1127, Paris, France
[11]Regional Cancer Institute Eugène Marquis, Rennes, France
[12]Radboudumc Nijmegen, The Netherlands
[13]UMCG, University of Groningen, The Netherlands
[14]BC Cancer Agency, Vancouver, BC, Canada
[15] Marseille, Université, APHM, Hôpital de la Timone, Marseille, France
[16]Institut Gustave Roussy, Villejuif, France
[17]Ospedale Bellaria, Bologna, Italy
[18]Brain Tumor Center and Department of Neurology, VU University Medical Center, Amsterdam, The Netherlands
[19]Medical University of Vienna, Vienna, Austria
[20]Haaglanden Medical Center, The Hague, The Netherlands
[21]Clinical Cooperation Unit Neurooncology; German Cancer Consortium (DKTK); German Cancer Research Center (DKFZ) and Department of Neurology and Neurooncology Program; National Center for Tumor Diseases; Heidelberg University Hospital; Heidelberg, Germany
[22]German Cancer Consortium (DKTK) and CCU Neuropathology German Cancer Research Center (DKFZ) and Department Neuropathology, Institute of Pathology, University of Heidelberg, Heidelberg, Germany.
[23]Malnati Brain Tumor Institute at the Lurie Comprehensive Cancer Center, Northwestern University Feinberg School of Medicine, Chicago, IL, USA
[24]Department of Radiation-Oncology (MAASTRO clinic), and GROW (School for Oncology) Maastricht University Medical Centre, The Netherlands
[25]Department of Radiation-Oncology, Paracelsus Clinic Osnabrück and University of Munster, Germany

Corresponding author : Monika.Hegi@chuv.ch

Supplementary Table S1

Table S1. Comparison of clinical base line characteristics between the patient populations of the EORTC 22033 clinical trial included or not-included in this study

Variable	Modality	Included	Not Included	Statitstic	P-value
N		132	345		
Gender[a]	Female	58	144	0.10992	0.7402
	Male	74	201		
Mean age in year (SD)[b]		43.67 (11.01)	44.96 (11.98)	-1.1098	0.2681
Type of Surgery[a]	Biopsy	18	171	52.716	<0.0001
	Partial resection	85	121		
	Total resection	29	52		
	n/a	0	1		
Treatment[a]	RT	69	171	0.18213	0.6696
	TMZ	63	174		
Molecular subtype cohort [a,c,d]					
	IDHwt	7	42	11.256	**0.004**
	IDHmut non-codel	60	105		
	IDHmut codel	43	61		
	missing	22	137		
HR for PFS in IDH mutant only (IC 95%)[e]	1p/19q codeletion	1.07 (0.5975-1.918)	**1.88 (1.138-3.107)**		

[a] Chi-squared test

[b] Whelch t-test

[c] 110/132 in this study overlap with the 318 in the molecular subtype cohort reported in Baumert et al. 2016 [1].

[d] Molecular classification as reported in the clinical trial results [1]

log-ranktest and Cox Regression for PFS.

p-value < 0.05 in bold

Legends to Supplementary Figures S1-S9

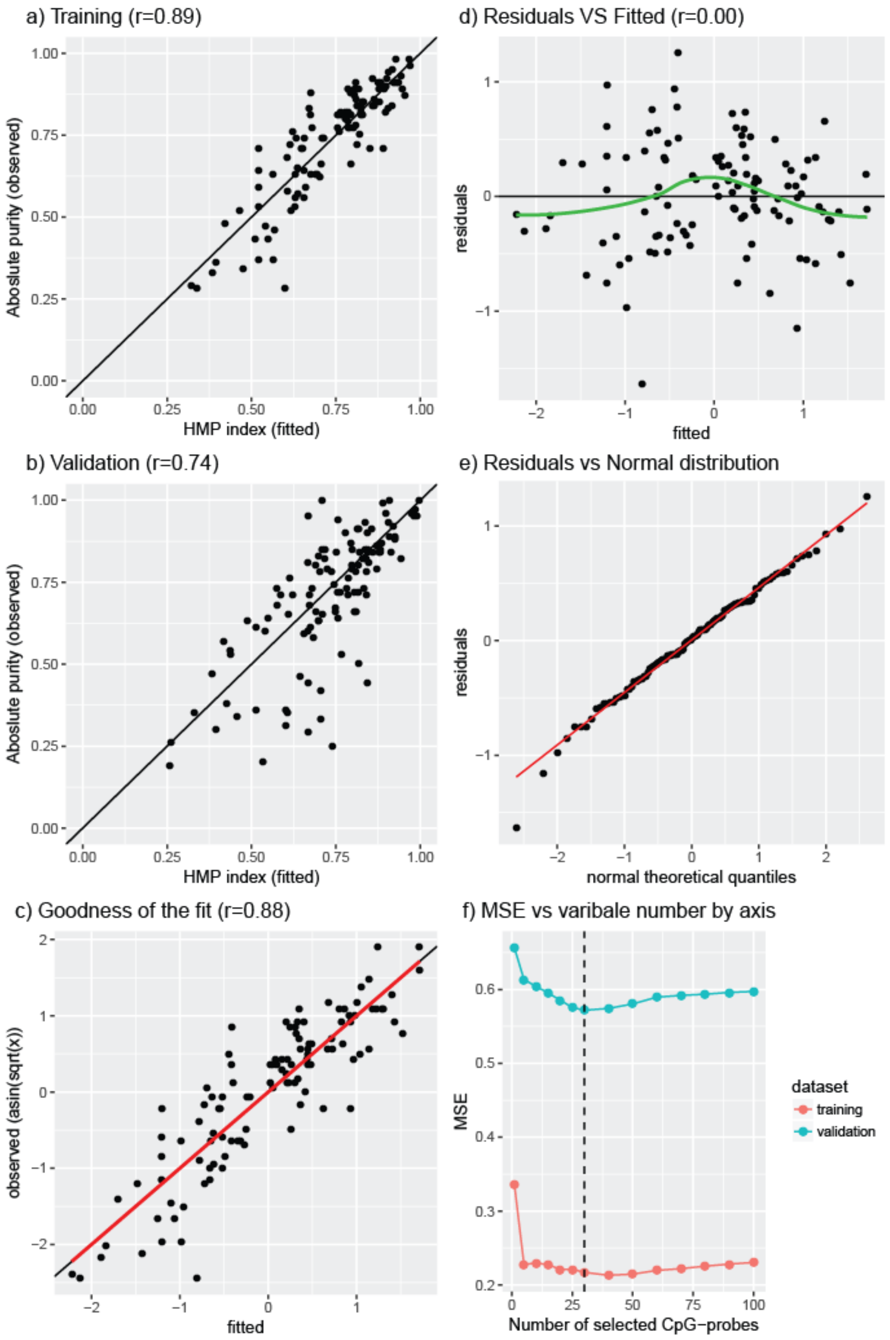

Supplementary Fig. S1 Predictive model for the purity index (HMP index) based on DNA methylation data from TCGA. The prediction of the purity of the samples was based on a model of Sparse Partial Least Squares (SPLS) with two components and unmethylated (β-median < 0.2) CpG-probes located in intergenic regions. The DNA methylation information was used to predict ABOSULTE purity estimation [2] after arcsin-square-root transformation. The SPLS regression used PLS-NIPALS

algorithm (maximize covariance between variable of interest and predictors) with lasso regularization [3]. One dataset from TCGA was used as training dataset and the second was used for validation. The performance of the model was evaluated by the comparison of Absolute purity estimation with the HMP index from the sPLS prediction for training (**a**) and validation (**b**) datasets. The goodness of fit is evaluated by the plot of observed values against fitted values (**c**). Graphic based on residuals in function of the fitted values (**d**). QQ-plot representation compares the residuals distribution with normal theoretical quantiles (e). The mean squared error (MSE) was used to define the selection variables by lasso regulation (30 CpG-probes by components) (**f**).

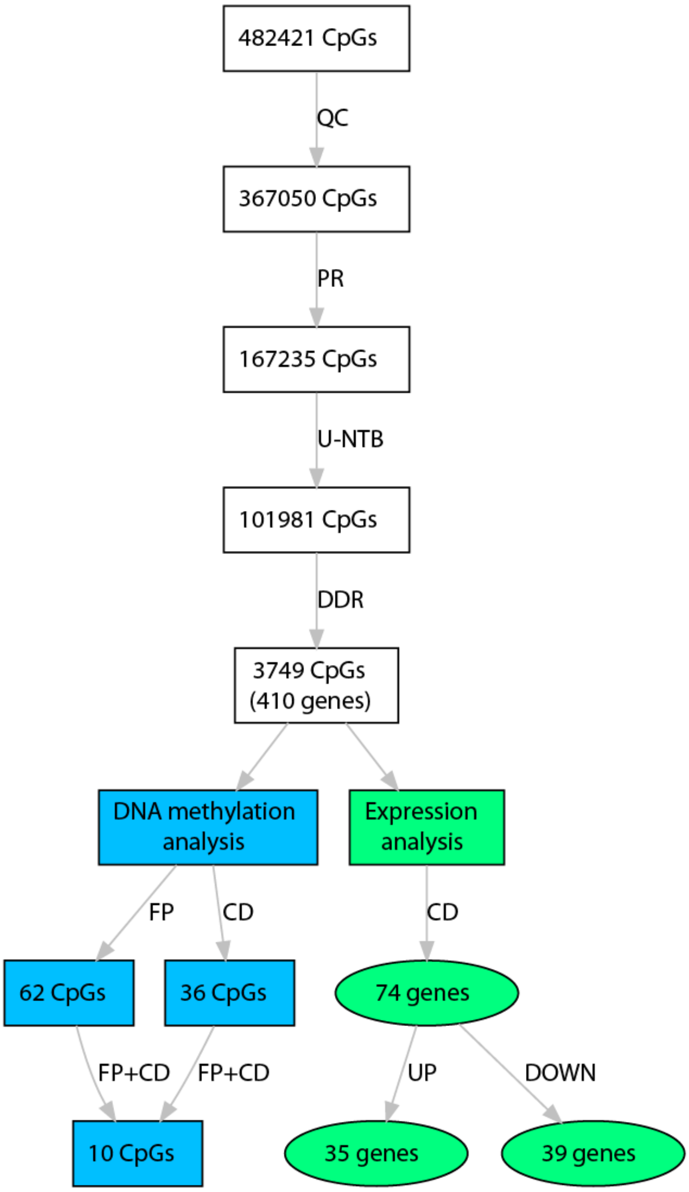

Supplementary Fig. S2 Flow chart diagram of the CpG (probe) and gene selection process. The flow chart of the gene selection process shows the steps through which we chose the final 62 functional DNA damage response (DDR) CpGs. There are 410 DDR genes represented on the HM-450K chip (after exclusion of CpGs on the sex chromosomes). DNA methylation and gene expression analyses correspond to the green and blue box respectively. The terms 'QC', 'PR','U-NTB' and 'DDR' refer to the quality control, promoter, unmethylated probe in non tumoral brain (NTB) and DDR gene selection steps, respectively. Functional selection, differential gene expression and differential DNA methylation between codeleted and non-codeleted groups were identified by the expressions 'FP' and 'CD'.

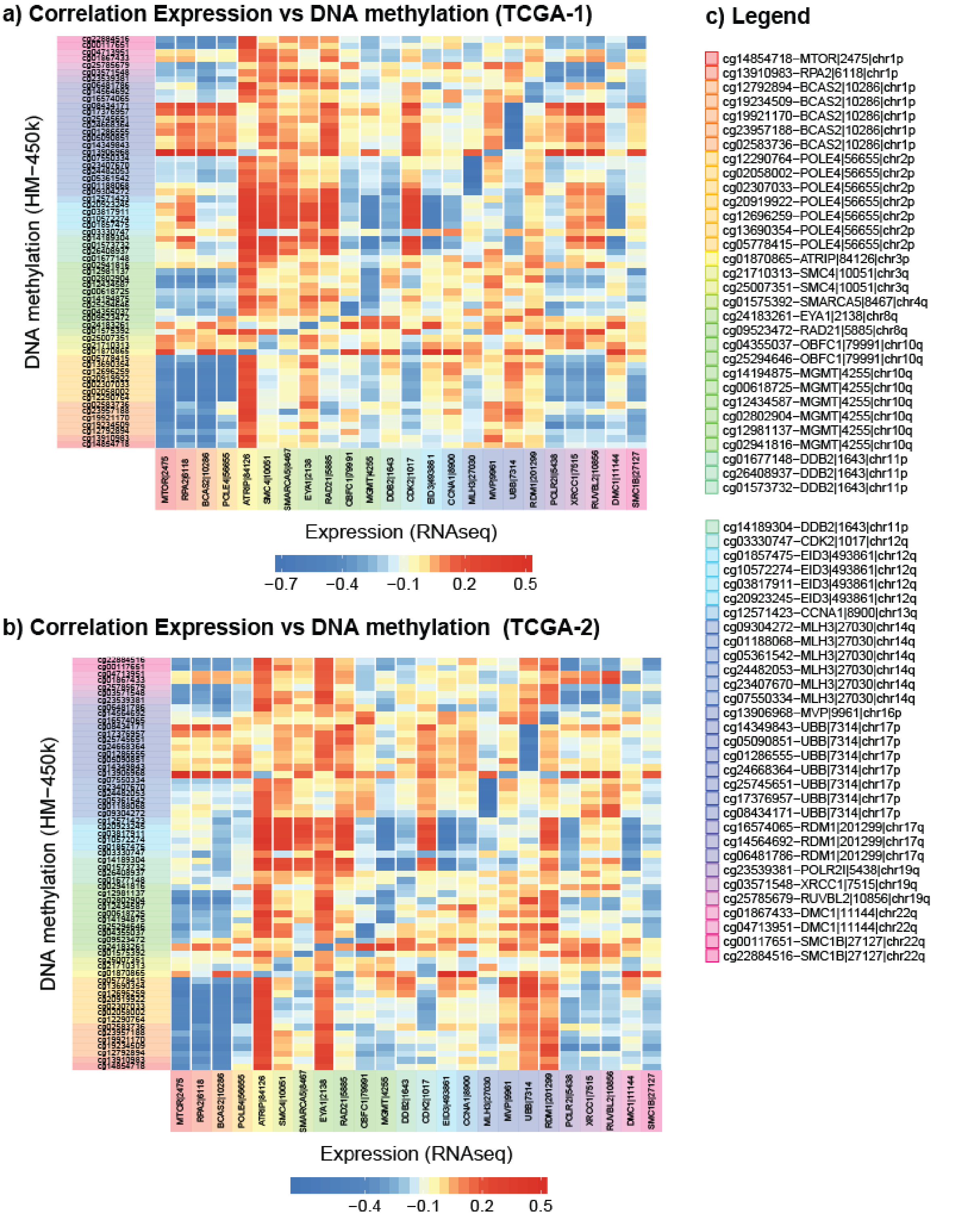

Supplementary Fig. S3 Correlation between DNA methylation and expression for the 62 functional DDR CpGs. The Pearson's correlations between CpGs and gene expression were calculated for the two TCGA datasets (TCGA 1 and TCGA 2) (**a**, **b**). The CpG probes are listed in (**c**) using the same color code as in **a** and **b**. The two datasets were highly similar as determined by comparing their Pearson correlation matrices between gene methylation and gene expression, illustrated in **a** and **b** (RV-coefficient = 0.92, P-value=0.001 for 999 permutations).

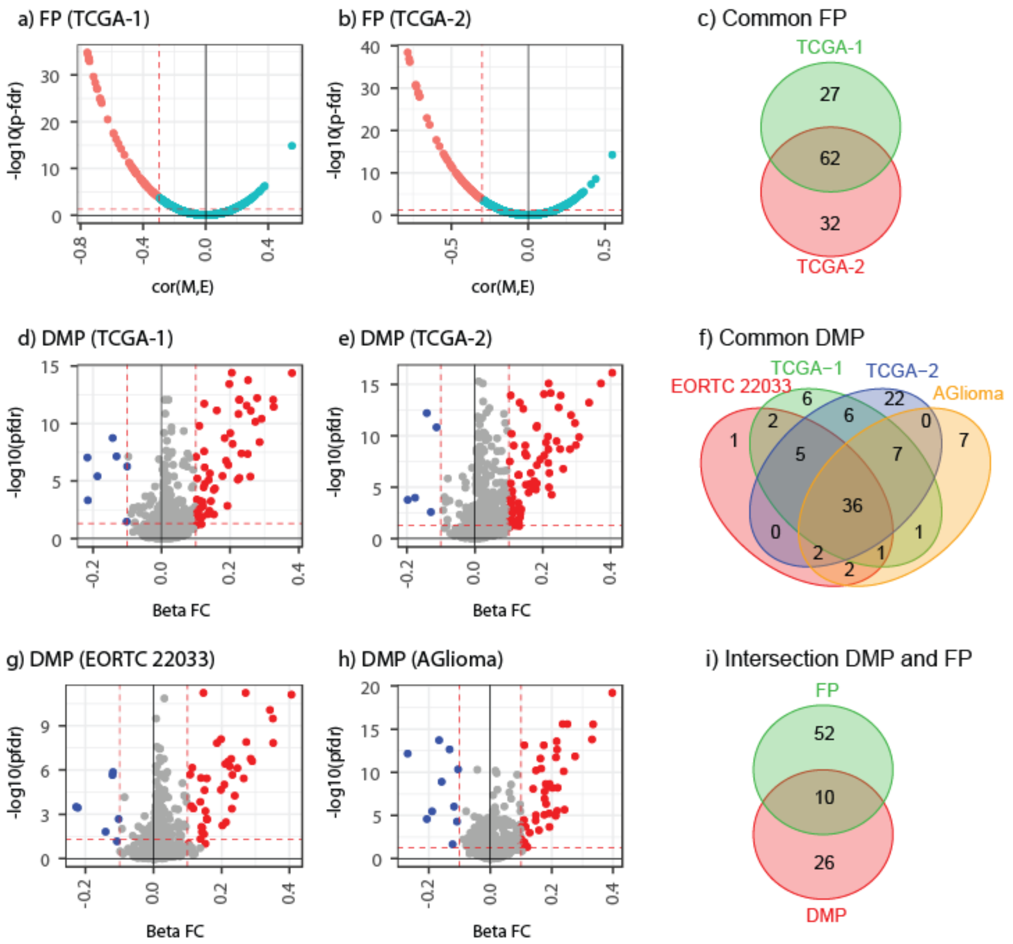

Supplementary Fig. S4 Functional methylation and differential methylation of DDR genes. Summary of the analyses identifying functionally methylated positions (FP) and differentially methylated positions (DMP) associated with DDR genes in TCGA datasets. The volcano plots based on the Pearson's correlation between expression and methylation (CpG) are used to detect FP for TCGA-1 (**a**) and TCGA-2 (**b**). The functionality of the CpG methylation (red points) is defined by a correlation inferior or equal to -0.3 and a p-fdr inferior or equal to 0.1. The volcano plot for differentially methylated positions between codeleted and non-codeleted IDHmt glioma is given for TCGA-1 (**d**) and TCGA-2 (**e**), EORTC-22033 (**g**) and AGlioma (**h**). The significantly hyper and hypo methylated CpGs in the codeleted group are identified by red and blue symbols, respectively. The dashed red lines correspond to two the cut-offs used for the selection of the candidate CpGs (p-fdr is inferior or equal to 0.1 and absolute value of fold-change for Beta-value is superior or equal to 0.1). The intersection between the analyses is illustrated by a Venn diagram for functional positions (**c**, 62 FP), differentially methylated positions (**f**, 36 DMP), and the intersection between FP and DMP (**i**, 10 CpGs). List of respective CpG probes is available in supplementary Table S2, Online Resource 2.

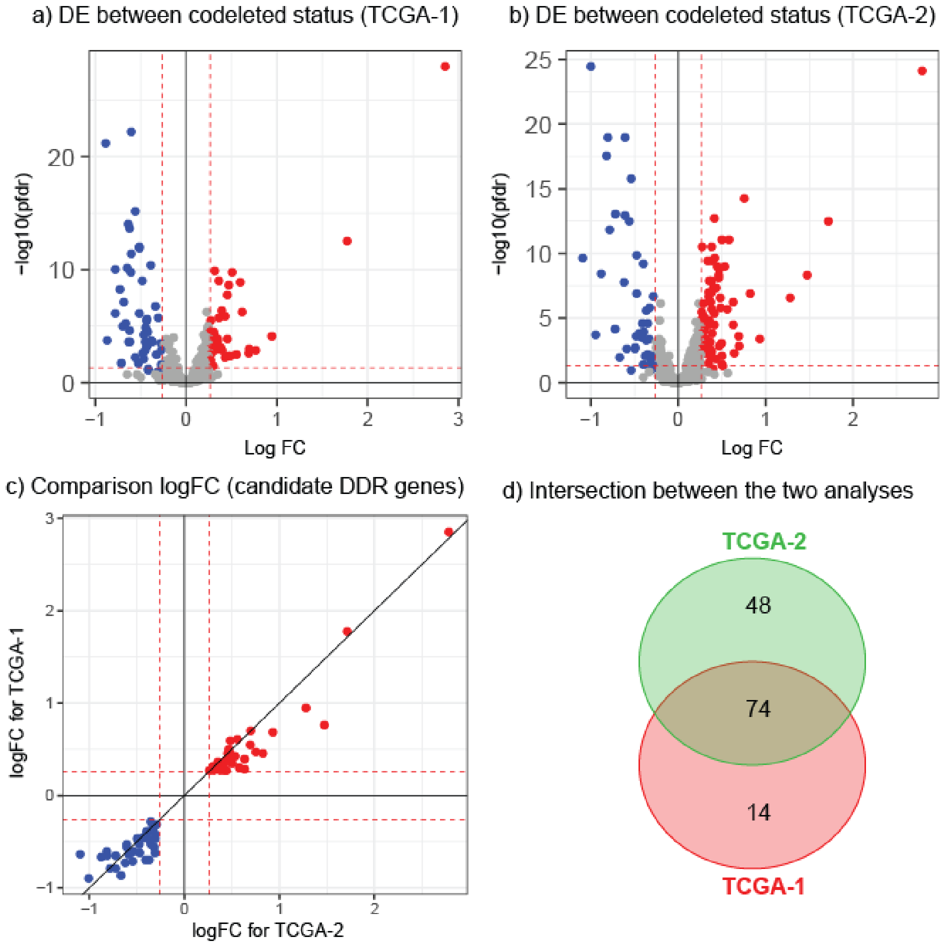

Supplementary Fig. S5 Differential DDR gene expression between codeleted and non-codeleted IDHmt glioma. Summary of differential expression (DE) analyses of DDR genes between codeleted and non-codeleted IDHmt glioma for the split datasets from TCGA. The volcano plots are given for TCGA-1 (**a**) and TCGA-2 (**b**) and show log fold change (Log FC) of gene expression (RNA-seq). The significantly up and down regulated genes in the IDHmt codeleted group are identified by red and blue symbols, respectively. The dashed red lines correspond to the cut-off used for the selection of the candidate genes (p-fdr is inferior or equal to 0.1, the absolute value of log2 fold-change is superior or equal to log2 (1.2)). The intersection between these two analyses is illustrated in the scatter plot (**c**) and the Venn diagram (**d**). Among the 74 detected genes, 39 genes were down-regulated (blue) and 35 were up-regulated in the IDHmt codeleted subpopulation. The list of the 74 DE genes is available in supplementary Table S3, Online Resource 3.

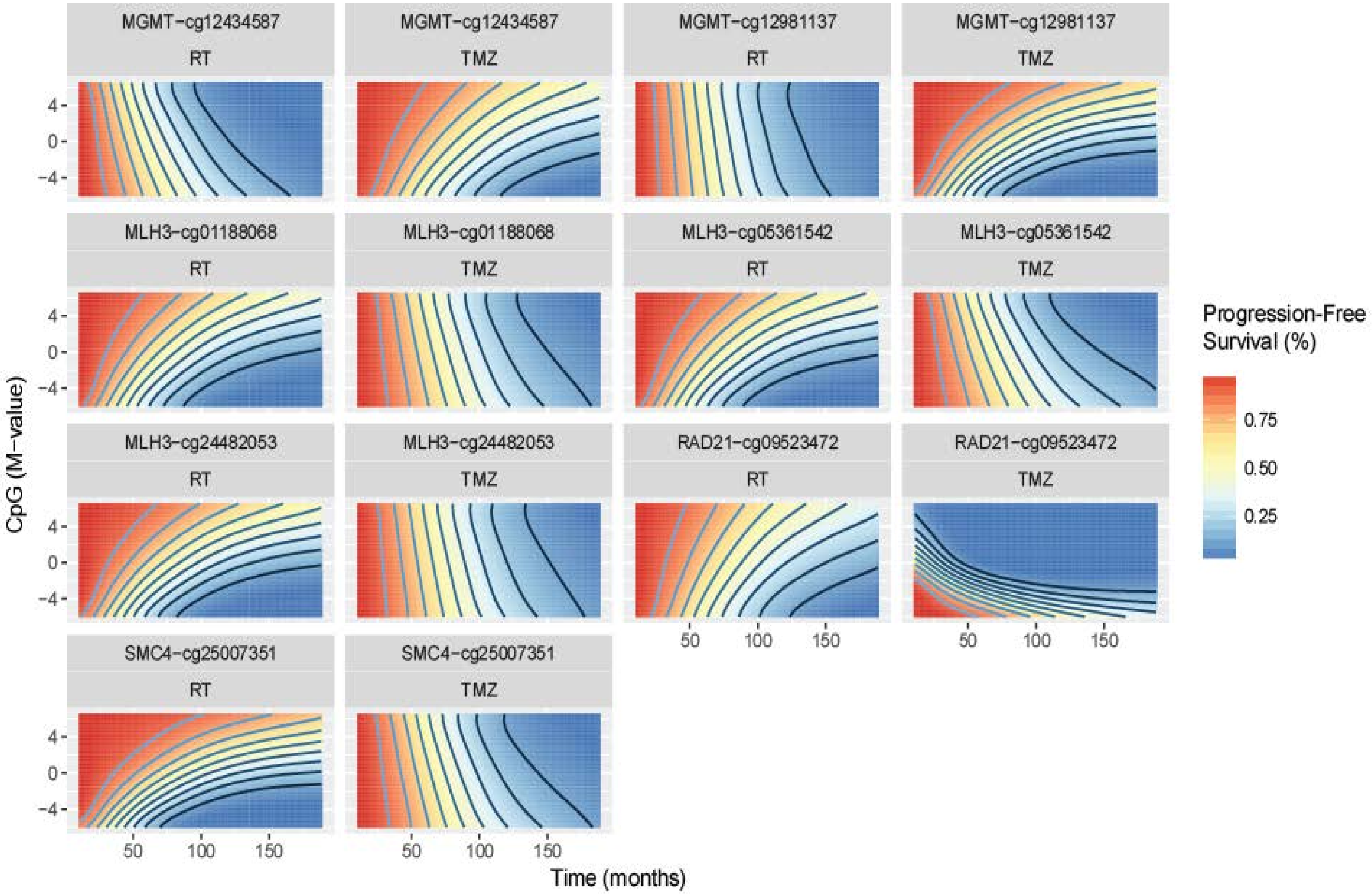

Supplementary Fig. S6 Simulation of progression-free survival (PFS) for functional DDR CpGs. The simulated progression-free survival (%, PFS) was based on the Cox Proportional-Hazards models for the 62 functional DDR CpGs. For the 14 CpGs with p-values <0.05 (not corrected for multiple testing) PFS is illustrated in function of time (month) for patients treated by radiotherapy (RT) and temozolomide (TMZ), respectively. Gradient color and contour lines provide the progression-free survival (%) .

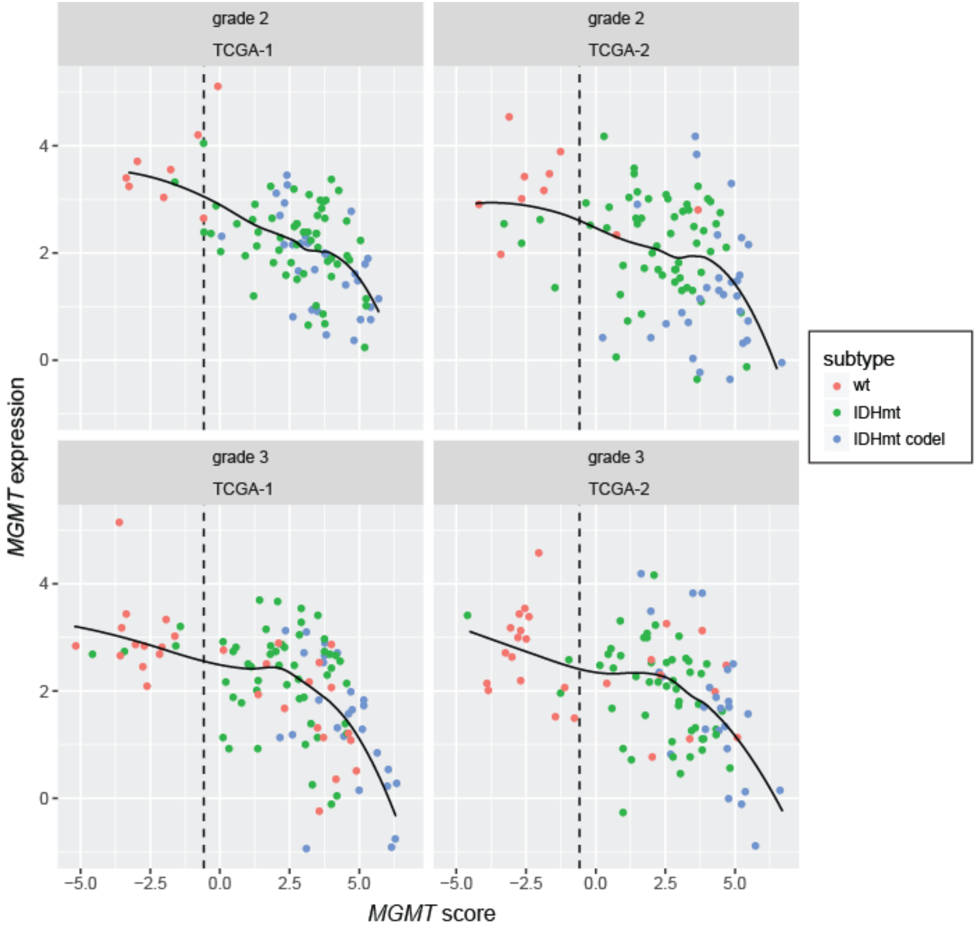

Supplementary Fig. S7 Representation of *MGMT* expression in function of the *MGMT* score in TCGA dataset. The association between *MGMT* expression (RNA-seq) and the *MGMT* methylation score is visualized for TCGA1 and TCGA2 stratified by WHO tumor grade. The wild-type (wt), the IDHmt non-codeleted (n) and codeleted (cd) samples are represented by dots colored in green, blue, and red, respectively. The trend is given by loess regression (black line) and the cut-off for the *MGMT* classification (MGMT-STP27) is represented by a dashed black line.

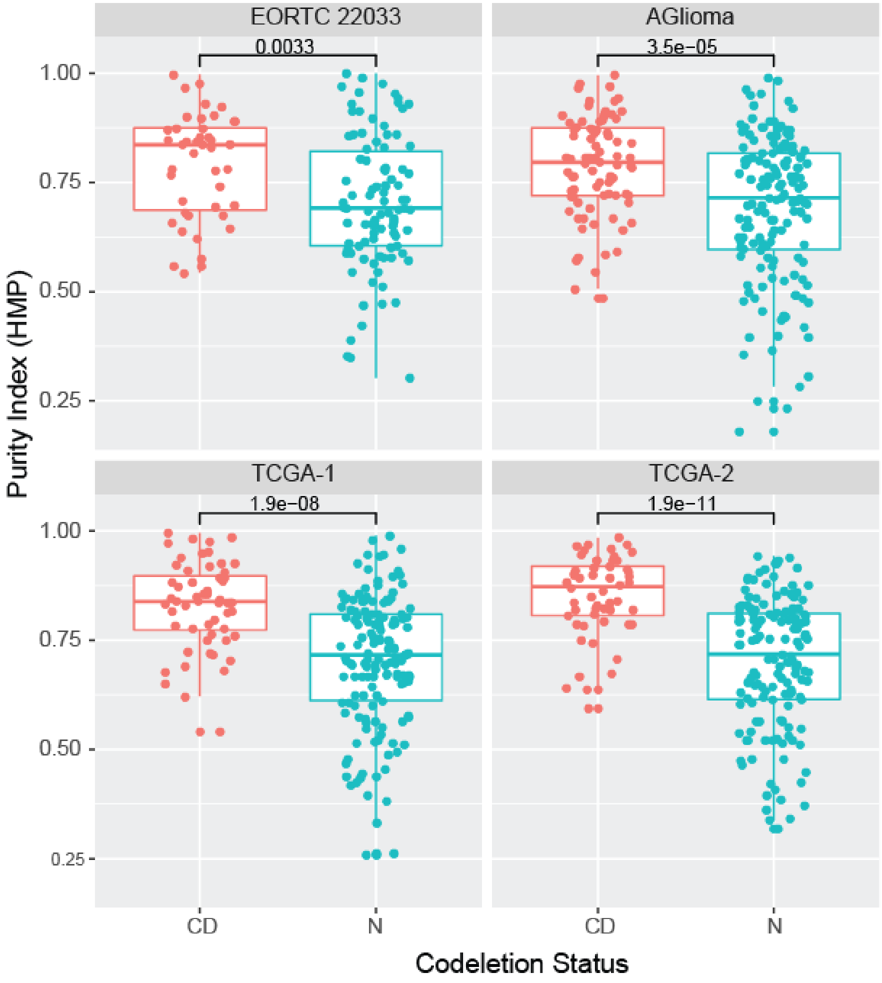

Supplementary Fig. S8 Boxplot representation of the purity index (HMP index) for codeleted and non-codeleted IDHmt glioma. The estimation of the purity is based on DNA methylation data from HM-450K (HMP index) in function of the molecular subtype (cd, codeleted or n, non-codeleted) by dataset. The codeletion status is significantly associated with the HMP index in all four datasets (p-value < 0.01 from Wilcoxon's test). The model for purity estimation is illustrated in supplementary Fig. S1.

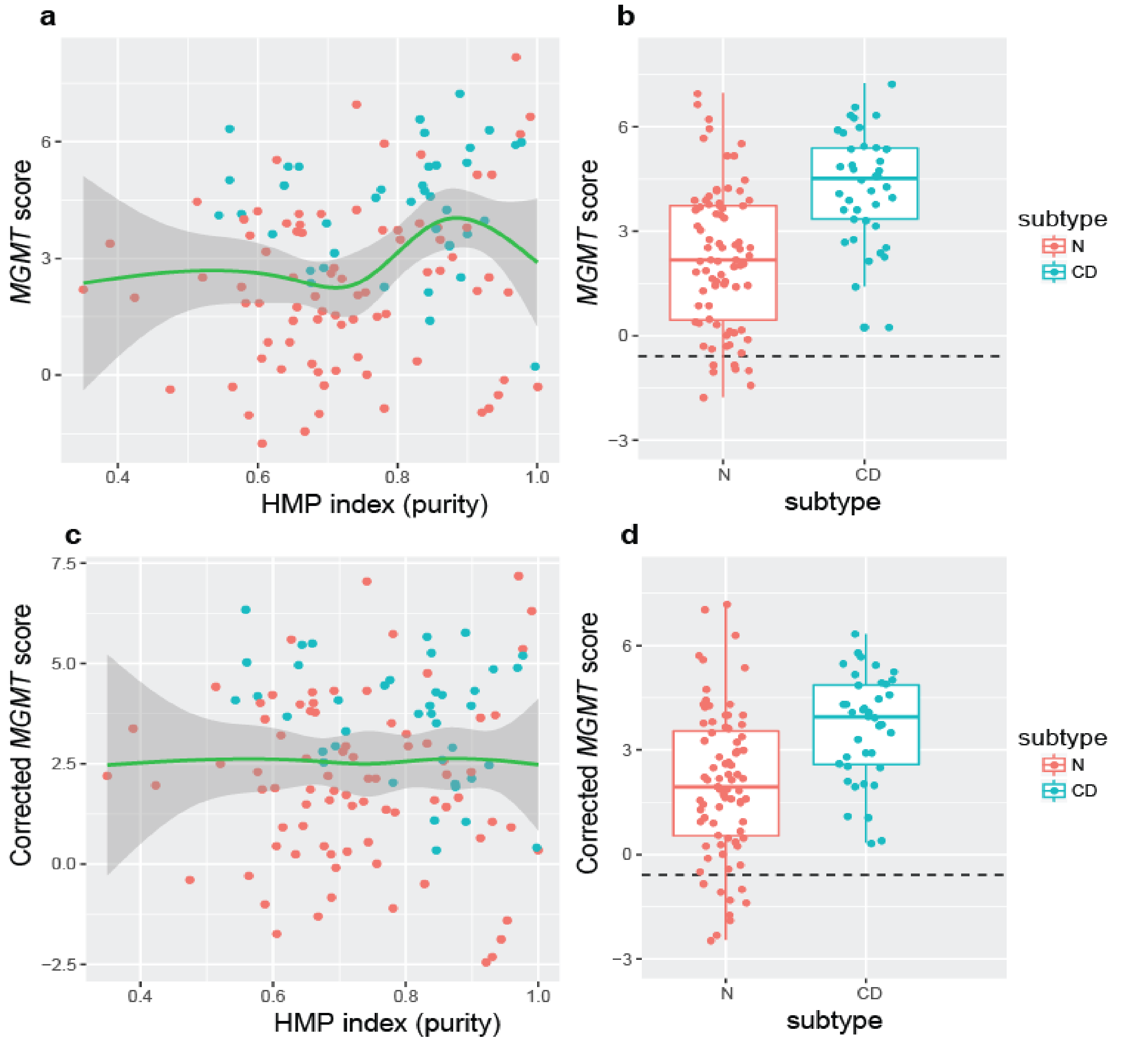

Supplementary Fig. S9. Relationship between *MGMT* score and purity index (HMP). The *MGMT* score and corrected *MGMT* score are represented in function of the HMP index (**a**, **c**) and in function of the molecular subtype (**b**, **d**). The corrected *MGMT* score was obtained after removing the purity effect (HMP index) using linear model and B-splines with four degrees of freedom illustrated by the green lines (**a**, **c**). The correction of the *MGMT* score by purity has a minor effect on the association of the *MGMT* score with the molecular subtype (**b**, **d**).

References:

1 Baumert BG, Hegi ME, van den Bent MJ, von Deimling A, Gorlia T, Hoang-Xuan K et al (2016) Temozolomide chemotherapy versus radiotherapy in high-risk low-grade glioma (EORTC 22033-26033): a randomised, open-label, phase 3 intergroup study. Lancet Oncol 17:1521-1532

2 Brat DJ, Verhaak RG, Aldape KD, Yung WK, Salama SR, Cooper LA et al (2015) Comprehensive, integrative genomic analysis of diffuse lower-grade gliomas. N Engl J Med 372:2481-2498

3 Le Cao KA, Rossouw D, Robert-Granie C, Besse P (2008) A sparse PLS for variable selection when integrating omics data. Stat Appl Genet Mol Biol 7:Article 35
